# Development of a M9‐based urea medium (M9U) for sensitive and real‐time monitoring of ureolytic activity of bacteria and cell‐free urease

**DOI:** 10.1002/mbo3.976

**Published:** 2020-01-14

**Authors:** Jens Jakob Sigurdarson, Simon Svane, Henrik Karring

**Affiliations:** ^1^ Department of Chemical Engineering, Biotechnology and Environmental Technology University of Southern Denmark Odense M Denmark

**Keywords:** ammonia, high throughput, inhibitor, plate reader assay, urease

## Abstract

The enzyme urease is widespread in nature and catalyzes the hydrolysis of urea to form ammonia and carbonic acid. The high proficiency of the enzyme is associated with a wide range of societal challenges. In agriculture, bacterial urease activity leads to loss of fertilizer through NH_3_ emission, which has a negative impact on the environment and human health. Urease is also an essential virulence factor for several pathogenic bacteria. To screen for potential urease inhibitors, efficient, sensitive, and accurate urease activity assays are needed. However, most urease activity assays are labor‐intensive and become time‐consuming when used to screen multiple samples. Based on systematic optimization, we have developed a urea‐containing growth medium and method for continuous real‐time monitoring and screening of urease activity from both bacterial cells and pure urease in a plate reader setup. The defined M9‐based urea (M9U) medium was found to be more sensitive and suitable for a plate reader setup than both Christensen's urea broth (CUB) and Stuart's urea broth (SUB), which are established and well‐known complex urea media that formed the principle foundation of M9U. Furthermore, we show that urease activity measurements using the M9U medium in our plate reader‐based method allow reliable high‐throughput screening of urease inhibitors.

## Graphical abstract

The enzyme urease is widespread in nature and catalyzes the hydrolysis of urea to form ammonia and carbonic acid. We have developed a M9‐based urea (M9U) growth medium and sensitive method for continuous real‐time monitoring and screening of urease activity from both bacterial cells and pure urease.

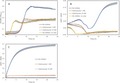

## 1 INTRODUCTION

The dinickel enzyme urease catalyzes the hydrolysis of urea, resulting in the overall formation of two ammonia molecules (NH_3_) and one carbonic acid molecule (H_2_CO_3_) per urea.(Mobley, Island, & Hausinger, 1995; Sigurdarson, Svane, & Karring, 2018) It is a common enzyme in nature and is found among plants, bacteria, algae, fungi, and invertebrates.(Bekheet & Syrett, 1977; Booth & Vishniac, 1987; Cook, 1976; Frankenberger & Tabatabai, 1982; Hanlon, 1975; Sumner, 1926) Urease in livestock animal feces is a concern in agriculture because it hydrolyzes the urea found in livestock animal urine, causing emissions of NH_3_ that can damage the environment, reduce air quality, and represent a loss of fertilizer nitrogen. (Sigurdarson et al., 2018) Urease is also an important virulence factor of several pathogens. The pathogenic ureolytic bacterium *Proteus mirabilis* can infect the urinary tract and cause complications such as the formation of kidney or bladder stones, which is a direct result of ureolytic activity. (Jones & Mobley, 1988; Mobley et al., 1995) Another pathogenic ureolytic bacterium is *Helicobacter pylori*, which infects the human gastric mucosa and can lead to stomach ulcers.(Mobley et al., 1995 *) H. pylori* can only survive because its urease creates NH_3_ to increase the pH in the surrounding otherwise acidic and harsh environment of the stomach (Krajewska, 2009).

To combat the environmental and health issues associated with the urease enzyme, it is important to have sensitive and reliable methods of studying urease activity and screening for potential urease inhibitors. The increase in pH following ureolysis can be exploited to determine whether an organism is ureolytic. In 1941, *R. Rustigian* and *C. A. Stuart* developed urea broth, or Stuart's urea broth (SUB), (Rustigian & Stuart, 1941) which is a complex growth medium designed to recognize ureolytic bacteria by incorporating both urea and the pH indicator phenol red. The color change of phenol red from yellow/orange at acidic conditions to red/pink at alkaline conditions occurs between pH 6.8 and 8.2. Thus, ureolytic bacteria growing in SUB at pH 6.8 will convert the urea into NH_3_, which will cause the pH to increase and the media to change color from yellow/orange to red/pink. In 1946, *W. B. Christensen* developed a different complex growth medium for identifying ureolytic bacteria based on the same principles.(Christensen, 1946) One of the changes that *W. B. Christensen* made was to reduce the concentration of phosphate buffer compared to that in SUB. By reducing the buffer capacity, less NH_3_ is required to overcome the buffer capacity of the medium and increase pH, thereby making the assay sensitive to lower levels of urease activity.

The dominant methods of quantifying and screening for urease activity have been to extract the enzyme and measure NH_3_ formation using the indophenol or nesslerization reactions. (Mobley & Hausinger, 1989; Sissons & Hancock, 1993) However, these are quite labor‐intensive methods and become time‐consuming if used to screen multiple samples, and they cannot be used for continuous real‐time monitoring of the urease activity. In this study, we report the development of a defined M9‐based and urea‐containing growth medium, M9U, and an efficient and sensitive pH‐based method to continuously measure urease activity from both bacterial cells and purified urease in real time utilizing a plate reader setup.

## 2 EXPERIMENTAL SECTION

### 2.1 Bacterial strains and urease enzyme

*Klebsiella pneumoniae* subsp. *pneumoniae* (ATCC: 13882, DSM No. 30102), previously known as *Klebsiella aerogenes*, was used as the urease‐positive bacterium for the experiments. *Escherichia coli* K12 MG1655 (ATCC: 700926, DSM No. 18039) was used as the urease‐negative bacterial control. Both bacterial strains were purchased from Leibniz Institute DSMZ‐German Collection of Microorganisms and Cell Cultures and were stored in 15% glycerol at −80°C.

Purified jack bean (*Canavalia ensiformi*s) urease was purchased from Sigma‐Aldrich and dissolved in an aqueous 15 mM KH_2_PO_4_ solution at pH 6.8 to give a final concentration of 1.89 mg/ml, corresponding to 66.15 U/ml. The urease stock was stored at −20°C.

### 2.2 Optimization of the M9‐based urea medium (M9U)

A new growth medium was developed for screening both bacterial and cell‐free urease activity. The medium is based on M9 minimal growth medium,(Miller, 1972) but with no Na_2_HPO_4_, a lowered KH_2_PO_4_ concentration of 2 g/L (14.7 mM), and the addition of 44.16 µg/L (0.34 µM) NiCl_2_, 12 mg/L (33.9 µM) phenol red and urea. To identify the optimal urea concentration in the medium, the M9‐based urea medium (M9U) was prepared with different urea concentrations: 0, 5, 10, 15, 20, 30, 40, and 50 mM. A microplate with wells containing either *K. pneumoniae* or jack bean urease was incubated for 24 hr at 37°C in the plate reader while shaking at 600 rpm with medium force. Absorbance was measured at 557 nm (max. absorbance for the deprotonated form of phenol red) and OD_630_ every 30 min. The growth curve of *K. pneumoniae* (expressed as OD_630_) was fitted to the Gompertz function as described by Zwietering, Jongenburger, Rombouts, and van 't Riet (1990) using the mathematical software Mathematica v.9.0.

### 2.3 Preparation of growth media

The majority of chemicals and ingredients used for media preparation were purchased from either Sigma‐Aldrich or Merck Millipore. All growth media and stock solutions were prepared using ultrapure water from an ELGA Purelab Chorus 1 system.

Christensen's urea broth (CUB) was prepared following the specifications presented previously. (Christensen, 1946) All components were mixed and autoclaved, except for glucose and urea, which were sterile‐filtered and added aseptically to the medium. SUB was prepared based on the recipe developed by *R. Rustigian* and *C. A. Stuart *(Rustigian & Stuart, 1941) and using a urea broth base (Sigma‐Aldrich 51463). After the medium was prepared, it was sterile‐filtered.

M9U was formulated using 2 g/L (14.7 mM) KH_2_PO_4_, 0.5 g/L (8.6 mM) NaCl, 0.012 g/L (33.9 µM) phenol red, 0.12 g/L (1 mM) MgSO_4_, 0.011 g/L (0.1 mM) CaCl_2_, 44.16 µg/L (0.34 µM) NiCl_2_, 0.5 g/L (9.3 mM) NH_4_Cl, 4 g/L (22.2 mM) D(+)‐glucose, 2.4 g/L (40 mM) urea, 2.3 mg/L (20 µM) FeCl_2_, 8.1 mg/L (50 µM) ZnSO_4_, and 10 ml/L of BME Vitamin solution 100X (Sigma‐Aldrich B6891). All M9U components except glucose, urea, FeCl_2_, ZnSO_4_, and vitamins were mixed, the pH adjusted to 6.8 and the medium was autoclaved. Glucose, urea, FeCl_2_, ZnSO_4_, and BME Vitamin solution were sterile‐filtered and added aseptically after autoclaving. For the three growth media, urea was added within 24 hr before the medium was used.

### 2.4 Plate reader experiments

Plate reader experiments were performed using a Varioskan LUX multimode microplate reader (Thermo Fisher Scientific) and transparent sterile F‐bottomed 96‐well microplates (Brand, pureGrade S, 781662). All microplates were sealed with optically clear heat seal tape (Thermo Fisher Scientific, AB‐0812 Diamond Seal) using an ALPS 30 heat sealer (Thermo Fisher Scientific).

For microbial urease activity experiments, *K. pneumoniae* and *E. coli* were grown overnight in the respective growth media. The cells were pelleted by centrifugation for 5 min at 16,000 × *g*, the supernatant removed, and fresh media added to reach an OD_600_ of 0.125 of the bacterial suspension, corresponding to a final OD_600_ of 0.05 in the well. To each well, 80 µl of bacterial suspension and 120 µl of growth media were added to reach a final volume of 200 µl. In the urease inhibition assays, 80 µl of bacterial suspension, 100 µl of growth media, and 20 µl of inhibitor solution were added instead.

In the cell‐free urease activity assays, the jack bean urease stock solution was diluted with 15 mM KH_2_PO_4_ solution at pH 6.8 to reach a concentration of 2.65 U/ml. For each well and 5 µl of diluted urease solution (2.65 U/ml) was added along with 195 µl of growth media, resulting in a final urease concentration of 0.07 U/ml. In the urease inhibition assays, 5 µl of enzyme solution, 175 µl of growth media, and 20 µl of inhibitor solution were added per well.

### 2.5 Comparison of urea‐containing growth media

The urea growth media CUB, SUB, and M9U were analyzed with both ureolytic *K.* *pneumoniae* and pure jack bean urease. The microplate was incubated in the plate reader for 20 hr at 37°C while shaking at 600 rpm with medium force. The absorbance at 557 nm and OD_630_ was measured every 30 min.

### 2.6 Urease inhibition assay

Stock solutions of 100 mM hydroxyurea (from Sigma‐Aldrich), 20 mM NBPT (N‐(n‐butyl) thiophosphoric triamide, from Carbosynth), and 7 mM PPDA (phenylphosphorodiamidate, from Fisher Scientific) were prepared and stored at −20°C. Each inhibitor was investigated in two concentrations. Thus, hydroxyurea was investigated at 10‐fold and 100‐fold dilutions of the stock solution, while NBPT and PPDA were tested at 10‐fold and 1000‐fold dilutions. All dilutions were analyzed with both *K. pneumoniae* cells and jack bean urease in microplates. The microplates were incubated in the plate reader for 24 hr at 37°C while shaking at 600 rpm with medium force. The absorbance at 557 nm and OD_630_ was measured every 15 min.

### 2.7 Statistical analysis

All results are expressed as the means of triplicate microplate wells with error bars showing the ± standard deviation (*SD*).

## 3 RESULTS

The advantage of using a defined medium over a complex medium when screening compounds for antiureolytic properties is that all specific constituents of the medium are known, and their concentrations can be altered accurately and independently. In contrast to a complex medium, where, for example, peptone and/or yeast extract contributes to the buffer capacity, a defined (minimal) medium normally only contains one buffer system. In addition, established complex urea media such as SUB and CUB have relatively high urea concentrations (0.67 M), which may affect the growth of the microorganism and the sensitivity of an assay.

### 3.1 Development of the defined urea growth medium (M9U)

**Figure 1:**
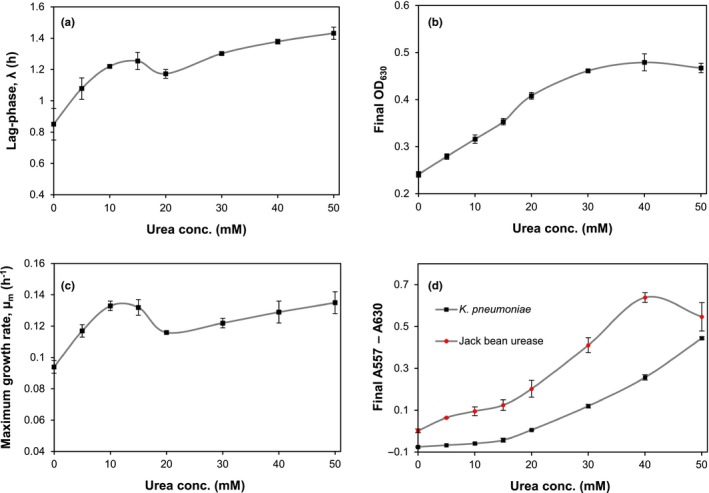
Effects of the urea concentration in M9‐based urea medium on the lagphase, maximum growth rate, stationary phase, and final pH. (a) the lagphase (λ) of *Klebsiella pneumoniae* related to the urea concentration. (b) the final OD_630_ reached by *K. pneumoniae* related to the urea concentration. (c) the maximum growth rate (µ_m_) of *K. pneumoniae* related to the urea concentration. (d) the final difference in absorbance at 557 nm and 630 nm (relates to the pH) caused by *K. pneumoniae* and jack bean urease related to the urea concentration

**Figure A1:**
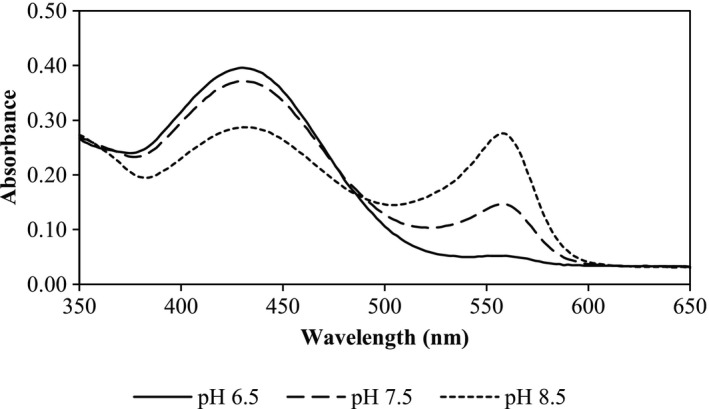
Absorbance spectrum of defined M9 growth medium with phenol red at pH 6.5, 7.5, and 8.5

**Figure A2:**
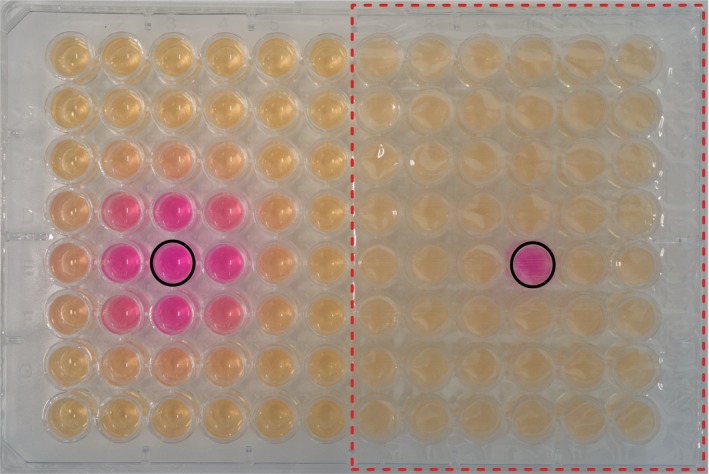
Microtiter plate showing the necessity of plate sealing to prevent contamination of neighboring wells due to ammonia volatilization. All wells contain CUB, and jack bean urease was added to one well on each half of the microtiter plate (wells marked with black circles). The right half of the microtiter plate is covered with sealing tape, as indicated by the red square. Several neighboring wells to the urease‐containing well (uncovered left half of the plate) are colored red/pink, most likely due to contamination of volatile ammonia produced from ureolytic activity

**Figure A3:**
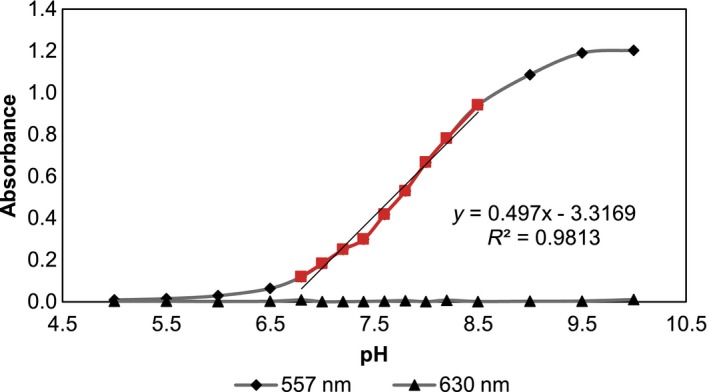
Linear relationship between pH and absorbance at 557 nm in M9U. The effect on absorbance at 557 nm and 630 nm when the pH of M9U is gradually increased. A linear fit and the corresponding R^2^ value are shown for the absorbance at 557 nm between pH 6.8 and 8.5 (indicated in red). The linear relationship can be described by the function A_557_ = 0.497 pH—3.3169

A medium suitable for detection and quantification of ureolytic activity was developed by modulating the well‐known M9 minimal growth medium (Miller, 1972) supplemented with nickel, urea, and phenol red. Thus, as for CUB and SUB, the pH increase caused by increasing ammonia concentration is followed using phenol red as a pH indicator. Using a plate reader that measures absorbance, it is possible to obtain a quantitative measure of the pH change. The absorbance spectra (350–650 nm) of M9 minimal growth medium with phenol red at pH 6.5, 7.5, and 8.5 showed increasing absorbance at 557 nm (deprotonated phenol red) and decreasing absorbance at 430 nm (protonated phenol red) as the pH increased (Appendix 1; Figure A1). When screening the urease activity of bacteria, it is necessary to take into account that cells will block or absorb light at all visible wavelengths. To obtain a true image of the pH‐induced absorbance change, it is thus necessary to subtract the optical density (OD) of the cells. Typically, OD is measured at 600 nm, but to prevent any interference from phenol red absorption, the OD was measured at 630 nm (Appendix 1; Figure A1). When using a ureolytic assay based on pH increase, it was found that it is essential to seal the microplate to avoid ammonia contamination between wells and, thus, false‐positive results (Appendix 2; Figure A2). This action, however, influences bacterial growth conditions, making the environment in the plate semi‐anaerobic. Consequently, the ureolytic bacteria that can be successfully screened are limited to facultative anaerobic bacteria. To identify the optimal concentration of urea, the effects of urea concentration on bacterial growth (lagphase (λ), maximum growth rate (µ_m_), and final optical cell density and pH change in the M9‐based urea medium were investigated (Figure 1a‐d). The lagphase of *K. pneumoniae* increases from approximately 0.85 hr to 1.25 hr when the urea concentration increases from 0 to 10 mM (Figure 1a). Above 10 mM urea, there is no significant effect on the lagphase. The maximum growth rate of *K. pneumoniae* is also affected by the urea concentration. Thus, between 0 and 10 mM, the growth rate increases and reaches a local maximum of 0.13/hr before it decreases. Between 20 and 50 mM, the growth rate increases again (Figure 1c). In particular, the final OD_630_ of *K. pneumoniae* is influenced by the urea concentration (Figure 1b). At concentrations up to 30 mM, the OD_630_ gradually increases and reaches a plateau at approximately 40 mM, indicating that nitrogen is no longer the growth‐limiting factor. The urea concentration also affects the final pH of the growth medium when *K. pneumoniae* reaches stationary phase (Figure 1d). The pH is stable up to approximately 15 mM urea, but above that concentration, the pH steadily increases. This result indicates that below 15 mM urea, the buffer capacity of the medium prevents any pH increase. Noteworthy, the decrease in the length of the lagphase and maximum growth rate at 15–20 mM urea coincide with the urea concentration at which the buffer capacity is breached, and pH increase starts to occur in the cell culture (Figure 1d); this observation indicates that the decreases in the length of the lagphase and maximum growth rate might be related to pH. For cell‐free urease, the pH continuously increases with increasing urea concentration until a maximum is reached at approximately 40 mM urea (Figure 1d). Based on these results, we concluded that it is generally best to use 40 mM urea in this defined M9‐based urea medium (M9U). Determination of the relationship between pH and absorbance at 557 nm in M9U (40 mM urea) showed a linear relationship within the range of pH values 6.8–8.5, which makes it easy to convert color (A557–A630) measurements to true pH values (Appendix 3; Figure A3).

### 3.2 Comparing bacterial growth and pH changes in different urea growth media

**Figure 2:**
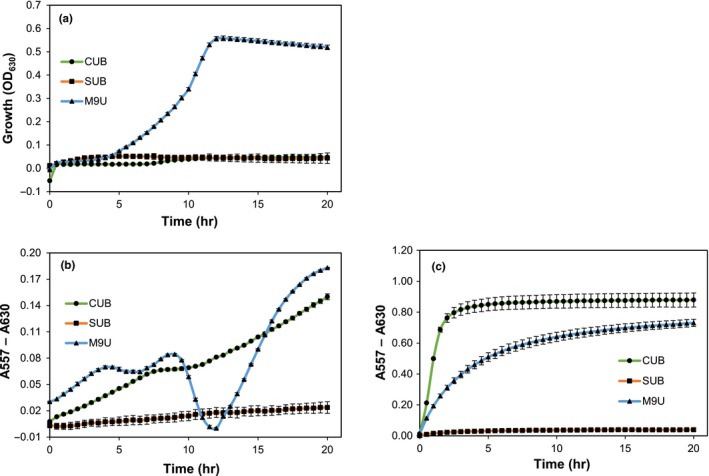
Comparison of growth and pH changes in three urea‐containing growth media, Christensen urea broth (CUB), Stuart's urea broth (SUB), and M9‐based urea medium (M9U). (a) growth of *Kebsiella pneumoniae* (measured as OD_630_). (b) change in pH (measured as a color change) caused by *K. pneumoniae*. (c) change in pH (measured as a color change) caused by purified jack bean urease

The new M9U growth medium was compared with the two most commonly used urea‐containing growth media, CUB and SUB. Both CUB and SUB were originally designed to qualitatively distinguish between *Proteus* spp. and other nonureolytic bacteria and, therefore, were not developed to quantitate urease activity in a microplate environment. However, the two media were compared here with M9U as they formed the basis for the development of M9U. In a microplate environment, M9U appears to provide better growth conditions for *K. pneumoniae* than the two complex urea growth media where little to no growth was observed (Figure 2a). The lack of growth is most likely due to the two media not being suitable for the microplate environment, where the sealing prevents exchange of air. Additionally, the more than 8‐fold higher urea concentration in CUB and SUB compared with M9U could play a role. It has previously been shown that increased urea concentration inhibits bacterial growth (Kaye, 1968). However, the maximum pH increase is quite similar between M9U and CUB, even though bacterial growth is much lower for the latter (Figure 2a,b). Thus, the results show that urease activity is still present despite the lack of growth. After approximately 10 hr, a significant drop in pH can be observed for *K. pneumoniae* in M9U (Figure 2b). The drop in pH occurs during the exponential growth phase and, therefore, could be caused by the production of acidic metabolites. When jack bean urease is used in the assay, pH reaches a higher level with CUB than with M9U, likely because CUB contains a higher urea concentration (Figure 2c).

### 3.3 Ureolytic activity in the presence of urease inhibitors

**Figure 3:**
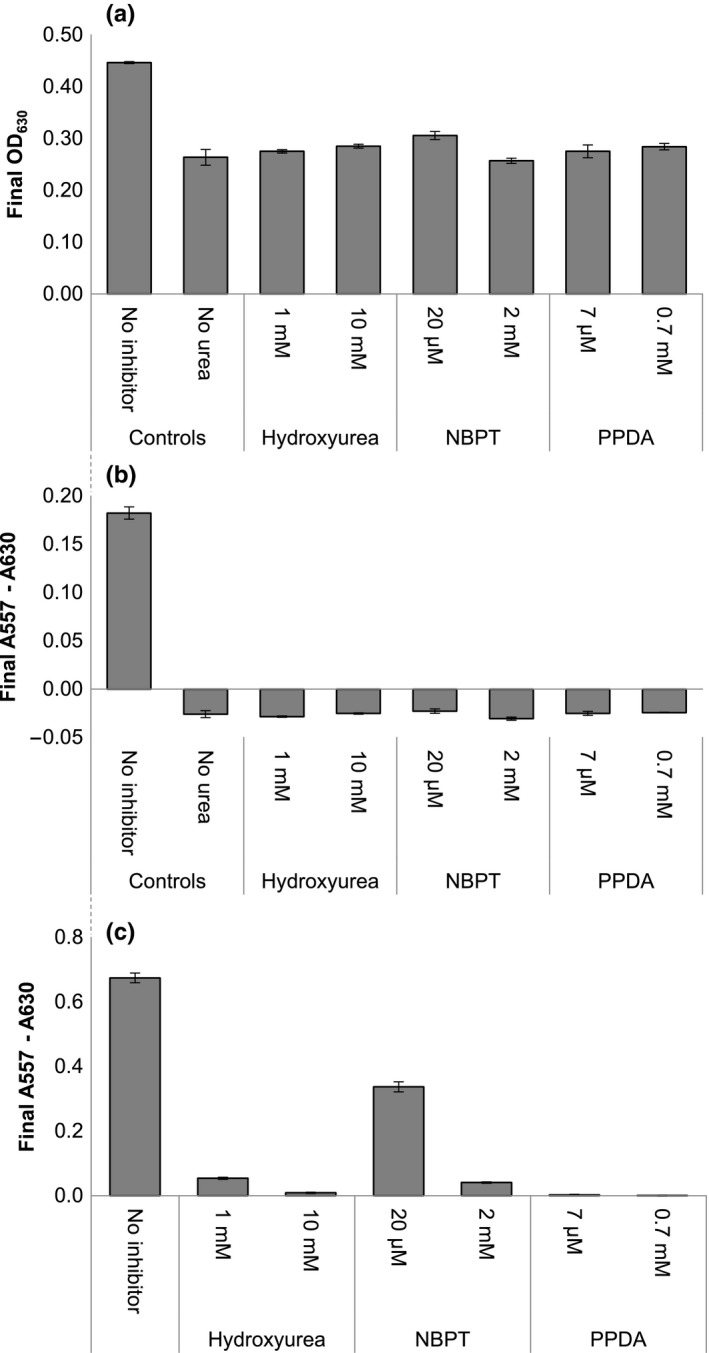
Determination of antiureolytic properties using M9U and the urease activity assay. Effects of the urease inhibitors hydroxyurea, NBPT, and PPDA on *Kebsiella pneumoniae* and cell‐free urease activity. (a) final optical cell density of *K. pneumoniae* (final OD_630_). (b) the final absorbance related to pH value (A557–A630) of the culture caused by *K. pneumoniae*. (c) the final absorbance related to pH value (A557–A630) caused by jack bean urease. Complete graphs from the experiments can be found in Appendix 4; Figures A4, A5, A6

**Figure A4:**
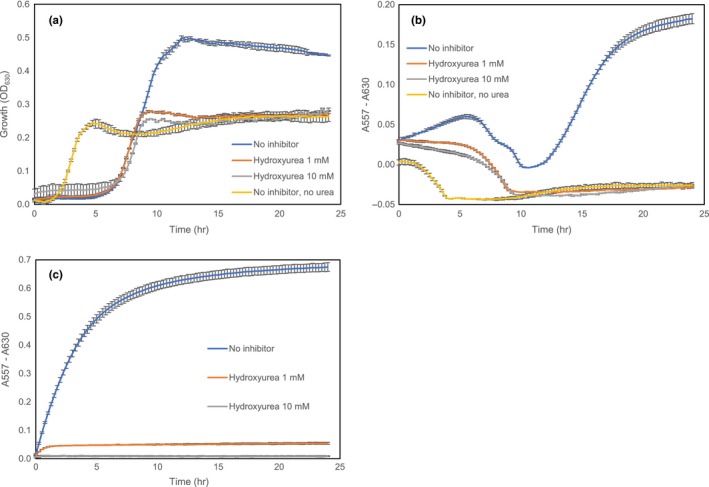
Effects of hydroxyurea on* K. pneumoniae *and cell‐free urease in M9U.A: growth curves of *K. pneumoniae *in M9U with 0 mM, 1 mM, and 10 mM hydroxyurea and one M9U control without hydroxyurea and urea. B: the change in absorbance related to the pH (A557–A630) of *K. pneumoniae* cultures in M9U with 0 mM, 1 mM, and 10 mM of hydroxyurea and one culture control without hydroxyurea and urea. C: the change in absorbance related to pH (A557–A630) caused by jack bean urease activity in M9U with 0 mM, 1 mM, and 10 mM of hydroxyurea. All curves are the means of triplicates with error bars showing the standard deviation

**Figure A5:**
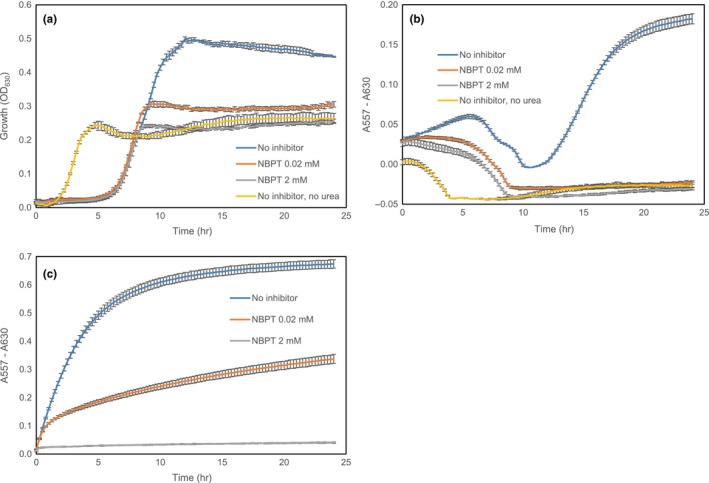
Effects of NBPT on* K. pneumoniae* and cell‐free urease in M9U.A: growth curves of *K. pneumoniae* in M9U with 0 mM, 0.2mM, and 2mM NBPT and one M9U control without NBPT and urea. B: the change in absorbance related to the pH (A557–A630) of *K. pneumoniae* cultures in M9U with 0 mM, 0.2mM, and 2mM of NBPT and one culture control without NBPT and urea. C: the change in absorbance related to pH (A557–A630) caused by jack bean urease activity in M9U with 0 mM, 0.2mM, and 2mM of NBPT. All curves are the means of triplicates with error bars showing the standard deviation

**Figure A6:**
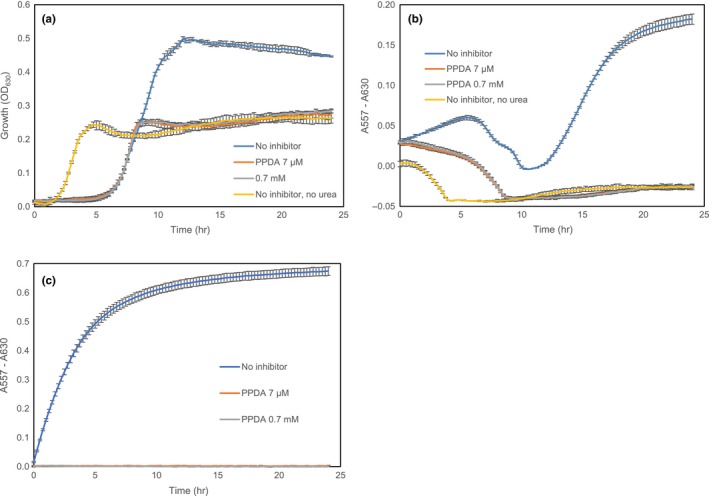
Effects of PPDA on* K. pneumoniae* and cell‐free urease in M9U.A: growth curves of *K. pneumoniae* in M9U with 0 mM, 0.007 mM, and 0.7 mM PPDA and one M9U control without PPDA and urea. B: the change in absorbance related to the pH (A557–A630) of *K. pneumoniae* cultures in M9U with 0 mM, 0.007 mM, and 0.7 mM of PPDA and one culture control without PPDA and urea. C: the change in absorbance related to pH (A557–A630) caused by jack bean urease activity in M9U with 0 mM, 0.007 mM, and 0.7 mM of PPDA. All curves are the means of triplicates with error bars showing the standard deviation

The potential of using the M9U and urease activity assay for screening compounds for antiureolytic properties were investigated with the urease inhibitors hydroxyurea, NBPT, and PPDA (Figure 3 and Appendix 4; Figures A4, A5, A6). For all inhibitors, the growth of *K. pneumoniae* is limited to the same extent as when grown without urea (Figure 3a). Additionally, in the presence of the inhibitors, the pH change is also reduced to approximately the same level as that observed for bacteria grown without urea (Figure 3b). It should be noted that when the bacteria are grown without urea or in the presence of the urease inhibitors, the final absorbance at 557 nm becomes negative because the M9U growth medium becomes more acidic relative to the starting pH (Appendix 4; Figures A4, A5, A6). This effect is likely due to acidic metabolites produced by the bacteria. The urease activity assay with purified jack bean urease in M9U containing urease inhibitors shows that PPDA completely inhibits urease activity, while the lowest concentration of NBPT only partly inhibits the cell‐free urease (Figure 3c).

## 4 DISCUSSION

**Figure A7:**
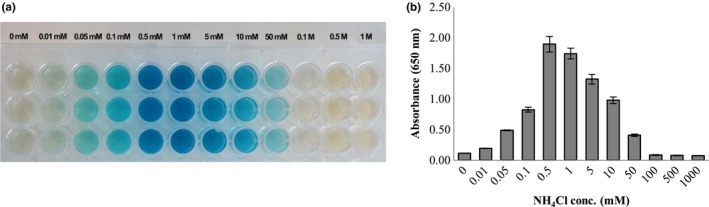
The indophenol assay can only determine ammonia in the μM range. A: microtiter plate with varying concentrations of NH_4_Cl (0 mM to 1 M), which have been analyzed using the indophenol method. B: absorbance measurements at 650 nm for the different concentrations of NH_4_Cl (0 mM to 1 M). All measurements are the means of triplicates with error bars showing the standard deviation

Plate readers have previously been used to determine urease activity. In a study by *R. L. Sinsabaugh *et al., urease activity in soil samples was assayed in a plate reader using the indophenol method to determine ammonia production.(Sinsabaugh, Reynolds, & Long, 2000) The indophenol method can be used to quantify urease activity, but is not suitable for real‐time measurements. In addition, our results show that the ammonia content can only be determined within a relatively narrow concentration range using the indophenol assay (Appendix 5; Figure A7). Furthermore, since the indophenol method is based on the reaction of chloroamine (produced by a reaction between HClO^‐^ and NH_3_) with phenols yielding blue‐colored indophenol, phenol functional groups in the growth medium components or inhibitor can potentially interfere with the assay. In 2013, *T. O. Okyay* and *D. F. Rodrigues* showed that urease activity from various ureolytic bacteria can be screened using SUB in a plate reader setup.(Okyay & Rodrigues, 2013) However, our results suggest that the microbial growth can be quite low in SUB, probably due to its high urea concentration (Figure 2). Additionally, the high buffer capacity of the SUB medium prevents the detection of low urease activity, decreasing the sensitivity of the assay.

Since urease activity depends on nickel atoms in the active site of the enzyme, nickel was added to the M9U medium to ensure that ureolytic bacteria in the assay are not limited in their synthesis of active urease. Previously, it was found that manure slurry from pigs contains 11.54 µM Ni^2+^, which is more than enough to maintain ureolysis.(Svane & Karring, 2019) However, to avoid toxicity/inhibition of urease, only 0.34 µM NiCl_2_ was included in the M9U medium. Recently, two new colorimetric methods to screen for urease activity in M9 media with a plate reader setup have been reported. (Amar, Peretz, & Gerchman, 2017; Tarsia et al., 2018) However, those studies focused on *H. pylori* urease using an engineered ureolytic *E. coli* strain. In addition, we have systematically optimized the defined M9 medium to make it suitable for sensitive and real‐time monitoring of the ureolytic activity of nonmodified bacteria or cell‐free urease. The simultaneous screening of urease activity from both a naturally ureolytic bacteria and cell‐free urease opens up the possibility of distinguishing between compounds directly inhibiting the enzyme and compounds preventing the production of active urease. Furthermore, the fact that we added only a low concentration of nickel to the defined urea‐containing medium makes it suitable for the screening of chelators that can bind nickel.

In summary, the ureolytic activity assay with the M9U medium is capable of screening cell‐based and cell‐free urease activity with a medium‐to‐high‐throughput setup. The assay distinguishes itself from other related methods by using an optimized defined M9‐based urea medium (M9U), which differs from other urea media based on M9 medium by having a lower buffer capacity, lower urea concentration, and lower nickel content, which altogether allow for increased sensitivity. Therefore, the assay is very applicable for the simultaneous screening of a large number of potential antiureolytic compounds using a high‐throughput setup.

## CONFLICT OF INTEREST

None declared.

## AUTHOR CONTRIBUTIONS

Jens Jakob Sigurdarson: Conceptualization‐Equal, Data curation‐Equal, Formal analysis‐Lead, Writing‐original draft‐Lead, Writing‐review & editing‐Equal; Simon Svane: Conceptualization‐Equal, Data curation‐Equal, Formal analysis‐Supporting, Writing‐original draft‐Supporting, Writing‐review & editing‐Equal;Henrik Karring:Conceptualization‐Equal, Formal analysis‐Supporting, Funding acquisition‐Lead, Supervision‐Lead, Writing‐original draft‐Supporting, Writing‐review & editing‐Lead.

## ETHICS STATEMENT

None required.

## Data Availability

The datasets used and/or analyzed during the current study are available from the corresponding author on reasonable request.

## Appendix 1

### Absorbance spectrum of M9 minimal growth medium with phenol red

#### Materials and methods

Normal M9 minimal growth medium with added phenol red was prepared. Briefly, the medium consisted of 22.2 mM glucose, 42.3 mM KH_2_PO_4_, 64.3 mM Na_2_HPO_4_, 18.7 mM NH_4_Cl, 8.6 mM NaCl, 1 mM MgSO_4_, 0.1 mM CaCl_2_, 18 µM FeCl_2_, 50 µM ZnSO_4_, 10 ml/l of BME Vitamin solution 100X (Sigma‐Aldrich B6891), and 33.9 µM phenol red. The growth medium was split into three portions, and the pH was adjusted to pH 6.5, pH 7.5, and pH 8.5 using dilute HCl and NaOH. Before preparing the M9 medium, all reagents were autoclaved apart from FeCl_2_, ZnSO_4_, glucose, and the vitamin solution, which were sterile‐filtered and later added aseptically to the medium. Subsequently, 200 µl of each of the three portions of each medium with different pH values was added to separate wells in transparent F‐bottomed 96‐well microplates (Brand, pureGrade S, 781662), and the full absorbance spectrum was determined using a Thermo Fisher Scientific Varioskan LUX multimode microplate reader.

#### Results

When the pH increased, the absorbance at 430 nm (corresponding to the yellow color of protonated phenol red) decreased, while the absorbance at 557 nm (corresponding to the red color of deprotonated phenol red) increased (Figure A1).

## Appendix 2

### Sealing microtiter plates with film to prevent ammonia volatilization

#### Materials and methods

The necessity of using a sealing film was investigated by adding 195 µl CUB to each well of a 96‐well microtiter plate. To one well on each half of the microtiter plate was added 5 µl of 2.65 U/ml jack bean urease (Figure A2). Half of the microtiter plate was then sealed with heat sealing tape (Thermo Fisher Scientific, AB‐0812 Diamond Seal), while the other half was left unsealed. Subsequently, the plate was incubated, with a lid, for 24 hr at 37°C while shaking at 600 rpm in a Grant‐bio Thermo‐shaker PHMP‐4.

#### Results

To avoid ammonia contamination between wells, and thus false‐positive measurements, it is essential that the microplate be sealed (Figure A2).

## Appendix 3

### Correlation between pH and the absorbance at 557 nm of the M9U medium

#### Materials and methods

The M9U growth medium was prepared as described in the main *Materials and methods* section and divided into 16 portions. The pH of the medium portions was adjusted to 5.0, 5.5, 6.0, 6.5, 6.8, 7.0, 7.2, 7.4, 7.6, 7.8, 8.0, 8.2, 8.5, 9.0, 9.5, and 10.0 using dilute NaOH and HCl. Subsequently, 200 µl of each portion of medium with different pH values was added to separate wells in a microtiter plate and the absorbance values at 557 nm and 630 nm were measured in the plate reader.

##### Results

There is a linear relationship between the pH of M9U and the absorbance at 557 nm, but only in the range between pH 6.8 and 8.5 (Figure A3).

## Appendix 4

### Urease inhibition assay

#### Materials and methods

See the main *Materials and methods* section of the article.

#### Results

Hydroxyurea, NBPT, and PPDA showed different effects on *K. pneumoniae* and cell‐free urease (Figures A4, A5, A6).

## Appendix 5

### Indophenol assay

#### Materials and methods

The measurement of increasing NH_4_Cl concentrations in a solution using the indophenol method was investigated in a microtiter plate setup. The indophenol method used was derived from the Danish Standard DS224 for determination of ammonia–nitrogen, though adapted to fit a microtiter plate volume. Briefly, 190 µl of NH_4_Cl solution was added to each well and mixed with 23 µl of Solution A (2.75 M sodium salicylate and 1.14 mM sodium nitroprusside) and 38 µl of Solution D (mixture of Solutions B and C), which was freshly prepared within 1 hr of the experiment. Solution D was made by mixing 5 ml of Solution B (2 M NaOH and 0.4 M citric acid) with 0.18 ml of Solution C (2 M NaClO). The mictrotiter plate was then incubated in the plate reader for 2.5 hr while shaking at 600 rpm before the absorbance was measured at 650 nm.

#### Result

NH_4_Cl concentrations below approximately 0.5 mM showed increasing absorbance with increasing amounts of NH_4_Cl (Figure A7). However, NH_4_Cl concentrations higher than 0.5 mM caused the absorbance at 650 nm to decrease (Figure A7). Consequently, it is essential that the sample of interest contain less than 0.5 mM NH_3_/NH_4_^+^ when using the indophenol method, which can otherwise lead to readings that are too low, causing significant NH_3_ measurement errors.
